# 4.6-billion-year-old aragonite and its implications for understanding the geological record of Ca-carbonate

**DOI:** 10.1007/s13146-015-0257-2

**Published:** 2015-08-13

**Authors:** Martin R. Lee, Paula Lindgren

**Affiliations:** School of Geographical and Earth Sciences, Gregory Building, Lilybank Gardens, Glasgow, G12 8QQ UK

**Keywords:** Aragonite, Carbonaceous chondrites, Marine mudrocks

## Abstract

Owing to its diagenetic instability, aragonite is rare in the geological record and almost entirely absent from pre-carboniferous sedimentary rocks. The former presence of this mineral in older deposits has to be inferred from petrographic, chemical or isotopic proxies. Crystals of aragonite that formed around 4563 million years ago occur in carbonaceous chondrite meteorites, showing that under certain conditions, the orthorhombic polymorph of Ca-carbonate can survive essentially indefinitely. Together with other carbonate minerals, phyllosilicates and sulphides, this aragonite formed by low-temperature water-mediated alteration of anhydrous minerals and glass in the interior of the meteorite’s parent asteroid(s). The survival of aragonite for such a long time can be attributed to the loss of free water by its incorporation into phyllosilicates, and to the very low permeability of the fine-grained and organic-rich rock matrix that prevented the ingress of fresh solutions via intergranular flow. By analogy with these meteorites, terrestrial aragonite is likely to survive where it has been similarly isolated from liquid water, particularly in organic-rich mudrocks, and such deposits may provide important new evidence for deducing the original mineralogy of skeletal and non-skeletal carbonates in deep-time.

## Introduction

Finding carbonate minerals in the geological record whose original crystal structure and composition have been preserved is crucial for understanding long-term changes to the Earth system including its climate and ocean chemistry. Correct determination of the mineralogy of biogenic carbonates is also important for tracking invertebrate evolution and studying taxonomic relationships. For example, secular variations in ocean chemistry have been identified through fluctuations between aragonite and calcite in the dominant mineralogy of inorganic marine carbonates (Sandberg 1975, 1983), and this record can be traced back 2700 million years (Hardie 1996, 2003; Ries et al. 2008). The relationships between the mineralogy and chemical composition of skeletal carbonates and seawater chemistry are considerably more complex owing to the biological control on mineralization and the operation of various evolutionary drivers (e.g., Kiessling et al. 2008; Zhuravlev and Wood 2008; Porter 2010). Using the calcium carbonate record to explore these and other processes in deep-time is challenging owing to the diagenetic instability of carbonate minerals in general, and aragonite in particular. Aragonite is scarce in Upper Palaeozoic sedimentary rocks (Wendt 1977), and there has been only one description of the mineral from Lower Palaeozoic deposits (Balthasar et al. 2011) and one from the Archaean (Lepot et al. 2008). The former presence of aragonite can only be inferred using proxies including the petrographic characteristics of replacive calcite and its chemical and isotopic compositions (e.g. Davies 1977; Mazzullo 1980).

The purpose of this short review is to draw attention to the oldest known aragonite, which occurs in carbonaceous chondrite meteorites and formed shortly after the birth of the Solar System. Although its occurrence in an extraterrestrial rock might imply that this aragonite is of limited use for understanding terrestrial carbonates, these meteorites are surprisingly similar in their properties to common lithologies on Earth. For example, the carbonaceous chondrites are comparable in grain size to mudrocks, and their original mineralogy and diagenetic history are analogous to that of basaltic volcanic ash. Furthermore, as the aragonite formed together with calcite and dolomite in an environment that was free of any biological influence, an understanding of the conditions under which it crystallised may suggest new directions for exploring inorganic carbonate precipitation on Earth.

## Carbonaceous chondrite meteorites

The carbonaceous chondrites are pieces of small planetary bodies, principally asteroids and possibly also comets, which have remained largely unchanged since the birth of the Solar System (McSween 1979). Eight groups of carbonaceous chondrites have been recognised, and are distinguished on the basis of their mineralogy, petrography, and chemical and isotopic compositions. Each group is a sample of one or more asteroidal or cometary ‘parent bodies’. The aragonite that is the subject of this review is most common in the CM group carbonaceous chondrites. The prefix ‘C’ denotes carbonaceous, and the suffix ‘M’ represents Mighei, which is the type meteorite of the CM group. The name Mighei comes from the place in the Ukraine where the meteorite was recovered in 1889 (Graham et al. 1985). All of the carbonaceous chondrites used in this study are described as ‘falls’, which means that they were seen to land and collected shortly afterwards. Thus, none of these meteorites have had the opportunity to interact with terrestrial water, and so we can be confident that the aragonite is extraterrestrial in origin.

Most of the CM carbonaceous chondrites contain chondrules, which are objects up to a few millimetres in size that are composed mainly of ferromagnesian silicate minerals. Chondrules formed at high temperatures within the solar nebula along with grains of silicate, oxide and sulphide minerals, metal and glass. The oldest of these objects, which are called calcium- and aluminium-rich inclusions, have been dated to 4568.2 + 0.2/−0.4 Ma (Bouvier and Wadhwa 2010). The majority of the CM meteorites belong to petrologic type ‘2’. This classification means that the chondrules lie within a fine-grained matrix that contains other ‘high temperature’ grains together with organic matter (up to ~2 wt%), and minerals including serpentines, sulphides and carbonates that formed by low-temperature water-mediated alteration of the most reactive of the high-temperature constituents (e.g. Tomeoka and Buseck 1985; Zolensky et al. 1993; Sephton et al. 2003; Brearley 2006). The aqueous fluids responsible for alteration were generated by the melting of water ice, probably accompanying heating of the interior of the parent body by short-lived radioisotopes including ^26^Al (e.g. Grimm and McSween 1989). Despite significant changes to their original mineralogy, the CM carbonaceous chondrites have largely retained their initial chemical compositions (McSween 1979). This apparent contradiction is explained by alteration having taken place in a chemically near-closed system within which aqueous solutions were essentially static, probably owing to the very low permeability (10^−19^ to 10^−17^ m^2^; 0.1–10 μD) of the fine-grained matrix (Bland et al. 2009). This value of permeability is very similar to that of terrestrial mudrocks (Neuzil 1995).

## Carbonate minerals in the CM2 carbonaceous chondrites

**Fig. 1:**
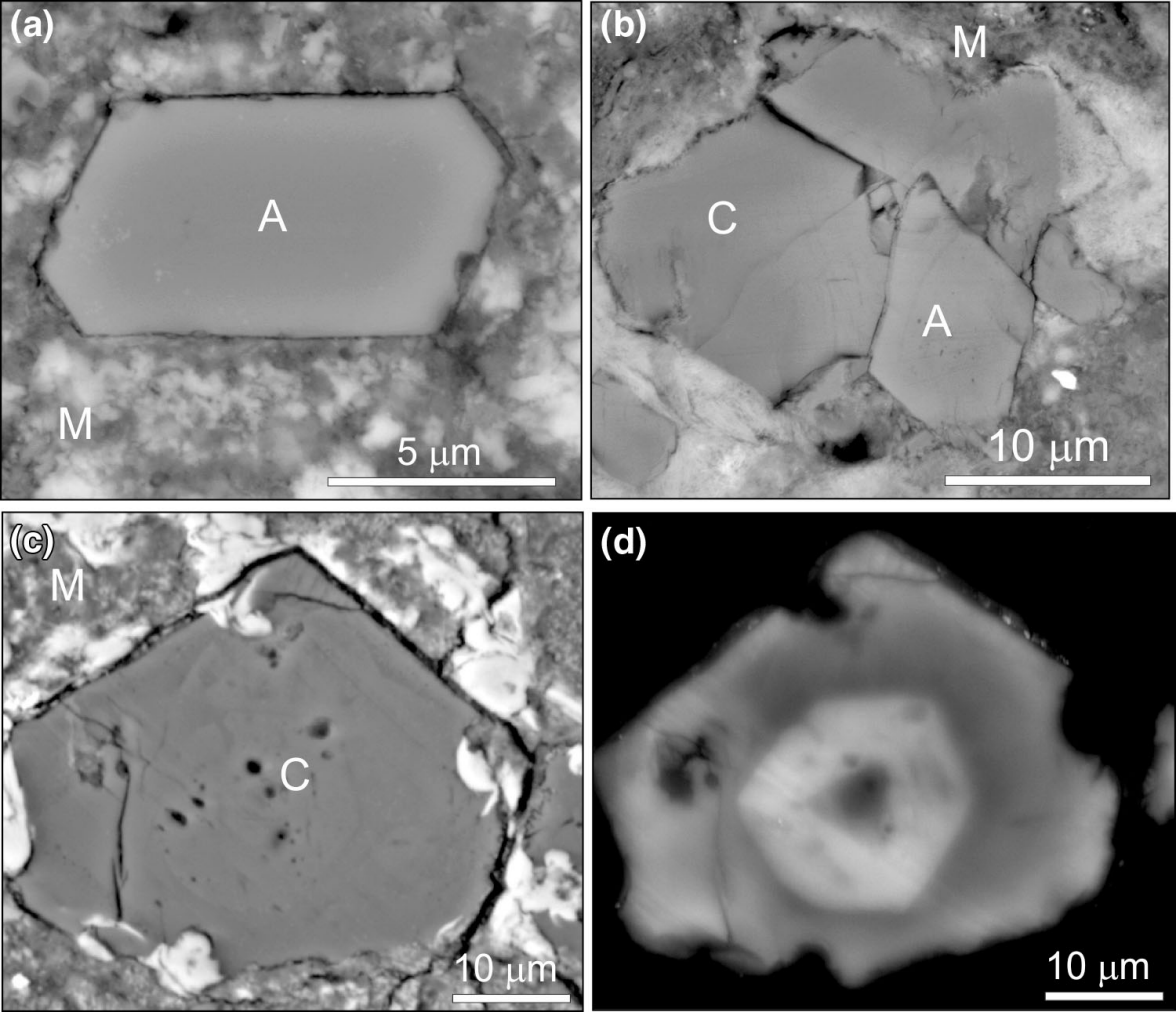
Images of aragonite (A) and calcite (C) in the fine-grained matrix (M) of CM2 carbonaceous chondrites. **a** Backscattered electron SEM image of a euhedral crystal of aragonite from the Murray meteorite. **b** Backscattered electron SEM image of the Murchison meteorite showing a crystal of aragonite enclosed within calcite. The* white-coloured* mineral surrounding the calcite is a hydrous sulphide. **c** Backscattered electron SEM image of a subhedral crystal of calcite in the Murray meteorite. **d** SEM-cathodoluminescence image of the same field of view as (**c**), showing that the calcite crystal is compositionally zoned

Carbonates constitute <~4 vol% of any one CM2 meteorite, and occur mainly in the fine-grained matrix. Four minerals have been identified: aragonite, breunnerite, low-Mg calcite and calcian dolomite (Ca_52–53_Mg_36–40_Mn_3–5_Fe_4–7_) (de Leuw et al. 2010; Lee et al. 2014). These minerals occur as single crystals or polycrystalline grains that range in size from ~5 to ~150 μm (Johnston and Prinz 1993; de Leuw et al. 2010; Lee et al. 2012) (Fig. 1). Aragonite has been distinguished from calcite by electron diffraction in the transmission electron microscope, cathodoluminescence (CL) spectroscopy, electron backscatter diffraction and Raman spectroscopy (Müller et al. 1979; Barber 1981; Lee and Ellen 2008; Lee et al. 2013). Element mapping and CL imaging reveal that crystals of all four minerals are commonly zoned (Fig. 1d), and the pattern of zoning suggests that they grew as cements in water-filled pores (e.g., Lee and Ellen 2008; Lee et al. 2014). Clumped isotope thermometry shows that calcite formed at temperatures of between 20 and 71 °C (Guo and Eiler 2007), which is consistent with temperature ranges obtained from considerations of matrix mineralogy (Zolensky et al. 1989). Grains of calcite and dolomite have been radiometrically dated using the ^53^Mn–^53^Cr system (^53^Mn decays to ^53^Cr with a half-life of 3.7 Ma), and results show that these minerals formed at 4563 + 0.4/−0.5 Ma (Fujiya et al. 2012), which is within ~5 Ma of the oldest objects in the Solar System (Bouvier and Wadhwa 2010). Thus, the carbonates are the products of a brief period of aqueous activity shortly after construction of their parent body by accretion within the solar nebula.

Aragonite crystals in the CM2 meteorites are commonly euhedral, and often elongate parallel to their *c*-axis (Fig. 1a). Some of them are enclosed within larger calcite grains, and the petrographic relationships between these two minerals show that the aragonite crystallised first (Fig. 1b); similar calcite overgrowths on aragonite have been described from vadose meteoric cements in the Bahamas (Curran and White 1995). The principal control on the polymorph of calcium carbonate that precipitated from aqueous solutions within the parent body was their Mg/Ca ratio. Specifically, those solutions in equilibrium with the aragonite had a Mg/Ca ratio of greater than 1, whereas solutions in equilibrium with calcite had a molar Mg/Ca ratio of less than 1 (Lee and Ellen 2008; Lee et al. 2014). Thus, the principal control on Ca-carbonate polymorphism in the asteroidal/cometary parent bodies of the carbonaceous chondrite meteorites was the same as in terrestrial seawater (Hardie 1996).

## Implications for exploring the terrestrial Ca-carbonate record

The presence of ~4.6-billion-year-old aragonite in the CM2 meteorites shows that despite the thermodynamic instability of this mineral at low temperatures and pressures (i.e. under the conditions prevailing in the interiors of asteroids and comets, and at shallow depths in the Earth’s crust), under certain conditions, it can survive essentially indefinitely. There are two reasons for its preservation in the meteorite parent bodies: (i) liquid water was present only briefly because it was consumed by the hydration of ferromagnesian silicates, metals and glasses to form phyllosilicates; (ii) the asteroids had a very low intergranular permeability owing to their fine grain size and organic matter content, which prevented any liquid water that may have been present elsewhere within the parent body from coming in contact with the aragonite.

Our explanation for the preservation of extraterrestrial aragonite is consistent with the occurrences of aragonite in low-permeability Phanerozoic rocks on Earth (e.g. Wendt 1977; Sandberg and Hudson 1983). For example, preservation of mollusc shells (originally aragonite) in the Pennsylvanian Buckhorn Asphalt was facilitated by early diagenetic invasion by hydrocarbons, which greatly reduced permeability (Seuß et al. 2009). Hallam and O’Hara (1962) described aragonite in mollusc shells from the Scottish Mississippian, and suggested that their preservation was due to the fine grain size of their host organic-rich mudrocks. A similar explanation was proposed for sub-micrometre-sized aragonite crystals that were described from an Archaean (2724 Ma) stromatolite (Lepot et al. 2008). In this latter case, preservation was facilitated by enclosure of the aragonite in globules of carboxyl-rich organic compounds, and the permeability in the rock was limited by the presence of clay minerals within the stromatolite layers.

Results from the meteorite record and from ancient terrestrial rocks together demonstrate that aragonite can be preserved essentially indefinitely where it is isolated from aqueous solutions by enclosure in a very low-permeability rock matrix. Thus, we predict that terrestrial marine mudrocks, especially those that are organic-rich, are likely to preserve the otherwise diagenetically unstable aragonite and high magnesium calcite. These rocks would therefore be attractive targets for future studies seeking to explore the evolution of the Earth system in deep-time.
